# Endophytic bacteria with plant growth promoting abilities from *Ophioglossum reticulatum* L.

**DOI:** 10.3934/microbiol.2017.3.596

**Published:** 2017-07-14

**Authors:** Ananya Mukherjee, Puja Bhattacharjee, Rituparna Das, Arundhati Pal, Amal K. Paul

**Affiliations:** 1 Microbiology Laboratory, Department of Botany, University of Calcutta, Kolkata, India; 2 Department of Botany, Serampore College, Serampore, Hooghly, India

**Keywords:** *Ophioglossum reticulatum* L., endophytic bacteria, enzymatic profile, antibiotic sensitivity, plant growth promoting traits

## Abstract

Endophytic bacteria colonizing the internal tissues of plants are known to improve plant growth by a wide variety of mechanisms. This study envisages the isolation and evaluation of plant growth promoting attributes of bacterial endophytes in perennial fern *Ophioglossum reticulatum* L. A total of 20 phenotypically distinguishable bacterial endophytes were isolated from surface sterilized leaf lamina, petiole, rhizome and spike of *O. reticulatum* L. The Shannon-Weaver diversity index showed that the rhizome (1.54) harbor more diverse types of endophytic bacteria than in its petiole, leaf lamina and spike. The isolated endophytes were characterized on the basis of micromorphological and physio-biochemical characters and tentatively assigned to the genus *Bacillus*, *Pseudomonas* and *Staphylococcus*. The isolates showed distinct variations in their enzymatic activities, sugar fermentation and antibiotic sensitivity profile. A number of endophytic isolates showed plant growth promoting activities like production of indole-3-acetic acid (IAA) and siderophore, growth in nitrogen-free medium and solubilization of phosphate. Time course of growth and IAA production by the potent isolate *Bacillus* OPR 7 have been determined. Exploitation of such plant growth promoting endophytes appears to be one of the best options in increasing biomass yield and improving plant fitness and productivity.

## 1. Introduction

All plants in nature harbor a diverse community of endophytic bacteria that colonize the internal tissues of the plant without imposing any negative impact on their host [1]. They have been isolated from roots, leaves, stems, flowers, fruits, and seeds from various plants [2] and found to play a pivotal role in plant growth enhancement. Production of phytohormones, solubilization of inorganic phosphate, sequestration of iron by siderophore, nitrogen fixation, etc. are the different ways by which endophytic bacteria stimulate plant growth [3],[4]. Such endophytic bacteria with plant growth promoting characters have been reported from different plants [5],[6]. These include species of *Pseudomonas, Azospirillum, Azotobacter, Klebsiella, Alcaligenes, Arthrobacter, Burkholderia, Bacillus* and *Serratia* [7]. *Enterobacter* spp. like *E. sakazakii* and *E.* *agglomerans* from soybean [8]; *E. cloacae* from citrus and maize [9],[10] and *E. asburiae* from sweet potato [11] have been reported to possess multiple plant growth promoting activities. *Planococcus* sp., *Micrococcus* sp*., Bacillus* sp., *Methylococcus* sp., *Acinetobacter* sp. and *Acetobacter* sp. endophytic to *Rosa damascena trigintipeta* were found to produce indole-3-acetic acid (IAA), solubilize calcium phosphate and produce siderophore [12].

In recent years, much attention has been focused on the natural methods of crop production for moving towards agriculturally and environmentally sustainable development. The use of bacterial endophytes as bio-fertilizers for improving crop production is gaining importance among agronomists and environmentalists as they would significantly reduce chemical input into the environment [13],[14]. The bacterial strain, *Bacillus* sp. SLS18 has been found to promote the biomass production of sweet sorghum [14], while the growth of poplar tree was increased by 60% after inoculation with different endophytic strains [15]. Both mycorrhizal fungi and bacterial endophyte have also been shown to enhance biomass production in switch grass [16],[17].

*Ophioglossum reticulatum* L. (Ophioglossaceae) is a small terrestrial erect fern, pantropical in distribution and is differentiated into a sub-terranean rhizome with roots and a single leaf bearing a simple, stalked, cylindrical sporangiferous spike with two rows of embedded sporangia. Out of the 40 species so far known, in India it is represented only by 12 species. However, unsustainable utilization is causing serious threat to the survival of a number of species. From economic view points young leaves are commonly used as salad or vegetable. Similarly, decoction of leaves and rhizomes are also used topically on boils, wounds and as an anti-inflammatory medicine. *Ophioglossum* spp. have been reported to be colonized by various species of vesicular arbuscular mycorrhizal (VAM) fungi, like *Endogone microcarpa, Enterophospora* sp., *Gigaspora* sp., *Glomus epigaeum, G. macrocarpum* and *G. occulatum* [18]. Such mycorrhizal association has been shown to improve plant health, disease resistance and drought tolerance. However, bacteria endophytic to *O. reticulatum* L. with plant growth promoting potential have not yet been reported. The aim of the present study was to isolate the endophytic bacteria from the surface sterilized leaf lamina, petiole, rhizome and spike of *O. reticulatum* L., characterize them to determine their taxonomic identity and to determine and evaluate their plant growth promoting activities.

## 2. Materials and Method

### 2.1. Collection of plant materials

*Ophioglossum reticulatum* L. (family Ophioglossaceae) plants with healthy leaves and mature spike were collected from Darjeeling hills, West Bengal (27°7′ N and 88°2′ E, 6710′ above sea level) during August–September, 2015–2016. Plants along with soil were collected in polythene bags, brought to the laboratory and stored at 4 °C until used for the isolation of bacterial endophytes.

### 2.2. Isolation and characterization of endophytes

Bacterial endophytes were isolated from the leaf lamina, petiole, rhizome and spike of healthy *O. reticulatum* L. The collected plant parts were first washed thoroughly under running tap water and transferred to sterile glass bottles for surface sterilization. The samples were sterilized by consecutive immersion in 70% ethanol (2–3 min), 0.5% sodium hypochlorite (5–10 min) and again with 70% ethanol for 30 sec. After washing for several times in sterile distilled water, the samples were cut into 2 mm sections and plated aseptically on previously prepared tryptic soy agar, glycerol asparagine agar and R2A agar plates for isolation of bacteria. The plates were then incubated at 32 °C for 2–4 days and observed for growth of the bacterial colonies surrounding the leaf lamina, petiole, rhizome and spike sections. Morphologically distinguishable bacterial colonies growing out of the plant segments were isolated in pure form by dilution streaking and maintained by regular sub-culturing on the same media. Bacterial strains were characterized and identified following micromorphological and physio-biochemical analysis following standard protocols.

### 2.3. Diversity of endophytes

Based on the total number of samples plated and the number of samples yielding isolates, the colonization frequency and the isolation rate were calculated. Colonization frequency of the bacterial endophytes was calculated as the total number of plant segments yielding the bacteria divided by the total number of segments incubated. Isolation rate was determined as the number of bacterial isolates obtained from the plant samples divided by the total number of samples incubated. The Shannon-Weaver diversity index was calculated as H = – Σ Pi ln Pi, where Pi is the species abundance.

### 2.4. Antibiotic sensitivity assay

Antibiotic sensitivity of the endophytic isolates was determined following the Kirby Bauer disc-diffusion assay method using antibiotic impregnated discs (6 mm dia., Himedia, India). Based on the diameter of inhibition zone recorded to nearest millimeter, the organisms were categorized as resistant, intermediate and sensitive following DIFCO Manual 10th edition (1984). Antibiotics used were penicillin G (1 Unit/disc), streptomycin (10 µg/disc), sulphatriad (300 µg/disc), tetracycline (25 µg/disc), ampicillin (10 µg/disc), and chloramphenicol (25 µg/disc).

### 2.5. Evaluation of plant growth promoting traits

#### 2.5.1. Indole-3-acetic acid production

The ability of the endophytes to produce indole-3-acetic acid (IAA) was determined following Salkowski colorimetric assay. Isolates were grown in tryptophan broth at 32 °C for 5 days and the culture filtrate was separated by centrifugation at 10,000 × g for 10 min. To 1 ml of the culture filtrate, 3 ml of Salkowski's reagent and 2 ml of distilled water was added. After an incubation of 30 min in dark, the tubes were observed for the development of pink colour. The OD was measured at 540 nm using a Systronics Photoelectric Colorimeter 112 and the quantity of the IAA produced was estimated from the standard curve prepared in the same way with authentic IAA from Sigma (USA).

#### 2.5.2. Phosphate solubilization

Ability of the bacterial endophytes for solubilizing insoluble phosphate was determined on Pikovskaya's medium supplemented with calcium triphosphate. The isolates were inoculated onto Pikovskaya agar and incubated for 5–7 days at 32 °C. The presence of halo zone around the bacterial colony was considered as an indicator for positive mineral phosphate solubilization. Solubilization index was calculated according to the ratio of the halo diameter to the colony diameter.

#### 2.5.3. Growth in nitrogen-free medium

Overnight grown bacterial endophytes were washed thoroughly in sterile normal saline and inoculated in Norris nitrogen-free medium. The ability of the isolates to fix atmospheric nitrogen was indicated by their growth in N_2_-free medium.

#### 2.5.4. Siderophore production

Production of siderophore by the endophytic bacterial isolates was tested qualitatively using chrome azurol S (CAS) agar following the protocol of Alexander and Zuberer [19]. The CAS agar, a mixture of four solutions, Solution 1 (Fe-CAS indicator solution), Solution 2 (PIPES buffer), Solution 3 (glucose, mannitol and trace elements) and Solution 4 (casamino acid) was prepared and sterilized separately before mixing. This mixture (Fe-CAS dye complex) yielded blue to dark green colour. The bacterial isolates were grown on it at 32 °C for 96 h. Orange halos around the colonies indicated siderophore production.

## 3. Results

### 3.1. Diversity of bacterial endophytes

**Figure 1.**
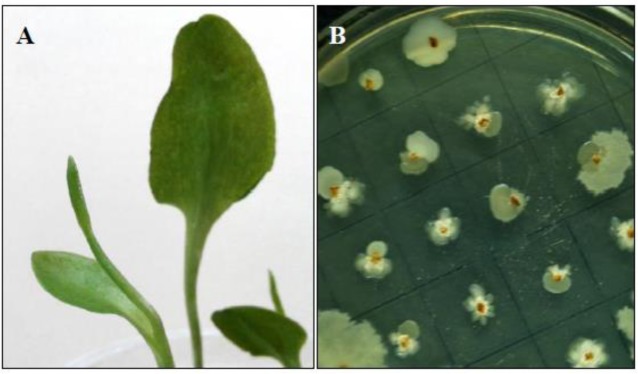
Mature *Ophioglossum reticulatum* L. plants with characteristic spike (A) and surface sterilized segments showing growth of characteristic bacterial colonies on tryptic soy agar plates (B).

Surface sterilized segments of leaf lamina, petiole, rhizome and spike of *Ophioglossum reticulatum* L. incubated on tryptic soy agar, glycerol asparagine agar and R2A agar plates showed growth of morphologically distinguishable bacterial colonies surrounding the segments after 48–96 h of incubation at 32 °C (Figure 1). Avoiding the repetitive strains, a total of 20 phenotypically distinguishable bacterial endophytes were isolated in pure form from 497 segments (202 leaf lamina, 60 petiole, 179 rhizome and 56 spike) of *O. reticulatum* L. Out of these 20 isolates, maximum (8) were derived from the rhizome and was followed by the leaf lamina (5), petiole (4), and the spike (3) tissues (Table 1). The colonization frequency was recorded to be low in petiole (35%) and spike (58.92%) samples as compared to leaf lamina (61.38%) and rhizome (87.7%). The isolation rate was poor in leaf lamina (0.02) but increased gradually in rhizome (0.04), spike (0.05) and petiole (0.06) samples. The Shannon-Weaver diversity index showed that the rhizome (1.54) of *O. reticulatum* L. harbor more diverse types of endophytic bacteria than in its petiole (1.05), leaf lamina (1.01) and spike (0.98).

**Table 1.** Diversity of endophytic bacteria in leaf lamina, petiole, rhizome and spike tissues of *O. reticulatum* L.

Parameters	Plant tissue				Total
	Leaf lamina	Petiole	Rhizome	Spike	
Number of samples used	202	60	179	56	497
Samples yielding endophytic isolates	124	21	157	33	335
Number of endophytic isolates	5	4	8	3	20
Colonizing frequency (%)^a^	61.38	35.00	87.70	58.92	67.40
Isolation rate^b^	0.02	0.06	0.04	0.05	0.04
Shannon-Weaver diversity index^c^	1.01	1.05	1.54	0.98	1.08

^a^Colonization frequency was calculated as the total number of plant samples infected by bacteria divided by the total number of samples incubated. ^b^Isolation rate was calculated as the number of bacterial isolates obtained from plant samples divided by the total number of samples incubated. ^c^Shannon-Weaver diversity index was calculated as: H = – Σ Pi X ln Pi, where, Pi is the proportion of individuals that species “i” contributes to the total.

### 3.2. Characterization and identification of the endophytic isolates

The bacterial endophytes of *O. reticulatum* L. were primarily characterized on the basis of micromorphological (Table 2) and physio-biochemical (Table 3) characters. Out of the 20 isolates 13 were Gram-positive (12 rods and a coccus) and 7 were Gram-negative rods. Almost all the isolates showed motility except OPL 4, OPP 4 and OPS 1. Endospore formation was observed in all the Gram-positive rods. Enzyme profile of the endophytes showed that while all the bacteria produced catalase, 95% of them produced caseinase and 85% produced gelatinase. Production of amylase (60%), inulinase (65%), PHB depolymerase (55%), lipase, CM cellulase and nitrate reductase (50%) were not uncommon. Glucose was utilized by all the isolates, while majority of the isolates could ferment glucose, sucrose and fructose (Table 4).

The micromorphological and biochemical characteristics (Tables 2–4) along with antibiotic sensitivity pattern (Table 5) of the endophytic bacterial isolates were compared with those described in Bergey's Manual of Determinative Bacteriology [20]. It was apparent that majority of the isolates (12 out of 20) could be assigned tentatively to the genus *Bacillus*. These include the isolates OPL 1, OPL 2, OPL 3, OPL 4, OPL 5, OPP 2, OPR 2, OPR 5, OPR 7, OPR 8, OPS 1 and OPS 3. The isolates OPP 1, OPP 3, OPR 1, OPR 3, OPR 4, OPR 6 and OPS 2 were tentatively placed under the genus *Pseudomonas.* Isolate OPP 4, the only Gram-positive, non-motile cocci was identified as *Staphylococcus* sp.

**Table 2.** Micromorphological characteristics of bacterial endophytes isolated from leaf lamina, petiole, rhizome and spike tissues of *O. reticulatum* L.

Plant part	Isolate	Colony morphology	Cell shape	Size, µm	Gram nature	Endospore formation	Motility
Leaf lamina	OPL 1	Cream, smooth, irregular	Rods, solitary or in chains of 4–6 cells	3.53–5.05 × 0.80–1.01	Gram + ve	+	+
	OPL 2	Cream, smooth, regular	Rods, mostly solitary, rarely in pairs	4.04–5.05 × 1.01	Gram + ve	+	+
	OPL 3	Yellow, smooth, irregular	Rods, solitary, often in chains of 4–6 cells	2.52–5.05 × 1.01	Gram + ve	+	+
	OPL 4	Cream, smooth, irregular	Rods, mostly in chains of 6–7 cells	3.03–4.04 × 1.01–1.2	Gram + ve	+	-
	OPL 5	Cream, smooth, irregular	Rods, mostly solitary or in pairs	3.03–4.04 × 1.01	Gram + ve	+	+
Petiole	OPP 1	Cream, smooth, irregular	Rods, mostly solitary or in pairs	3.03–5.05 × 0.20	Gram – ve	–	+
	OPP 2	White, smooth, irregular	Rods, mostly in pairs, sometimes solitary	3.03–5.05 × 0.80–1.01	Gram + ve	+	+
	OPP 3	Yellow, smooth, regular	Rods, mostly in groups, sometimes solitary	0.50–1.51 × 0.25–0.50	Gram – ve	–	+
	OPP 4	White, smooth, regular	Cocci, irregular groups of many cells	0.505–0.808 dia	Gram + ve	–	–
Rhizome	OPR 1	Cream, smooth, irregular	Rods, mostly in pairs, sometimes solitary	2.02–4.04 × 0.50	Gram – ve	–	+
	OPR 2	Cream, smooth, regular	Rods, mostly solitary	1.51–2.02 × 0.50	Gram + ve	+	+
	OPR 3	White, smooth, irregular	Rods, mostly in chains of 3–7 cells	4.04–7.07 × 1.01–1.11	Gram – ve	–	+
	OPR 4	Cream, rough, irregular	Rods, mostly in pairs, rarely solitary	3.03–5.05 × 0.50–1.01	Gram – ve	–	+
	OPR 5	Cream, smooth, regular	Rods, mostly solitary	3.03–4.04 × 0.80	Gram + ve	+	+
	OPR 6	Cream, smooth, irregular	Rods, mostly solitary, sometimes in pairs	2.52–4.04 × 0.50–1.01	Gram – ve	–	+
	OPR 7	Cream, smooth, irregular	Rods, mostly solitary	3.03–4.04 × 0.80	Gram + ve	+	+
	OPR 8	Cream, smooth, irregular	Rods, mostly solitary, sometimes in pairs	4.04–5.05 × 1.01	Gram + ve	+	+
Spike	OPS 1	Cream, smooth, irregular	Rods, mostly in pairs rarely in chains	5.05–6.06 × 1.01–1.51	Gram + ve	+	–
	OPS 2	Yellow, smooth, regular	Rods, solitary or in pairs	2.02–4.04 × 0.50	Gram – ve	–	+
	OPS 3	Brown, smooth, regular	Rods, in chains of 2–4 cells, or solitary	3.03–5.05 × 1.01–1.51	Gram + ve	+	+

**Table 3.** Biochemical characteristics of bacterial endophytes isolated from leaf lamina, petiole, rhizome and spike tissues of *O. reticulatum* L.

Plant part	Isolate	Production of enzyme									
		Catalase	Amylase	Caseinase	Gelatinase	Nitrate reductase	Cellulase	Lipase	Inulinase	Pectinase	PHB depolymerase
Leaf lamina	OPL 1	+	+	+	+	+	+	+	+	+	–
	OPL 2	+	+	+	–	–	–	–	+	+	–
	OPL 3	+	–	+	+	–	–	–	–	–	+
	OPL 4	+	+	+	+	–	+	+	+	–	+
	OPL 5	+	–	+	+	+	–	+	–	–	+
Petiole	OPP 1	+	+	+	+	+	–	+	+	+	+
	OPP 2	+	+	+	+	+	+	+	+	+	–
	OPP 3	+	–	+	–	+	+	–	+	+	+
	OPP 4	+	–	+	+	–	+	–	+	–	+
Rhizome	OPR 1	+	+	+	+	+	+	+	+	–	+
	OPR 2	+	+	+	–	–	–	+	–	–	–
	OPR 3	+	+	+	+	–	–	+	+	+	+
	OPR 4	+	+	+	+	+	–	–	–	–	–
	OPR 5	+	–	+	+	–	+	–	–	–	–
	OPR 6	+	–	–	+	–	+	–	–	–	–
	OPR 7	+	+	+	+	+	–	+	+	+	+
	OPR 8	+	+	+	+	+	+	–	+	+	+
Spike	OPS 1	+	+	+	+	+	–	–	+	+	–
	OPS 2	+	–	+	+	–	+	–	–	–	–
	OPS 3	+	–	+	+	–	–	+	+	–	+

“+” indicate positive response, “–” indicate negative response.

**Table 4.** Utilization and fermentation of sugars by bacterial endophytes isolated from leaf lamina, petiole, rhizome and spike tissues of *O. reticulatum* L.

Plant tissue	Bacterial isolate	Glucose		Sucrose		Fructose		Maltose		Lactose	
		U	F	U	F	U	F	U	F	U	F
Leaf lamina	OPL 1	+	–	+	–	+	–	+	+	+	–
	OPL 2	+	+	+	+	+	+	+	+	–	–
	OPL 3	+	+	–	–	+	+	+	–	+	–
	OPL 4	+	+	+	+	+	+	+	–	+	–
	OPL 5	+	+	+	+	+	+	+	+	+	+
Petiole	OPP 1	+	+	+	+	+	+	+	+	+	+
	OPP 2	+	+	+	–	+	–	–	–	+	+
	OPP 3	+	+	+	+	+	–	+	–	+	–
	OPP 4	+	+	+	+	+	+	+	+	+	+
Rhizome	OPR 1	+	+	+	+	–	–	+	+	+	+
	OPR 2	+	+	+	+	+	–	+	–	+	–
	OPR 3	+	+	+	+	+	+	+	+	+	+
	OPR 4	+	+	+	+	+	+	+	+	+	–
	OPR 5	+	+	+	+	+	+	+	+	+	–
	OPR 6	+	+	+	+	+	+	+	–	+	–
	OPR 7	+	+	+	+	+	+	+	+	+	+
	OPR 8	+	+	+	+	+	+	+	–	+	–
Spike	OPS 1	+	+	+	+	+	–	+	+	+	+
	OPS 2	+	–	+	+	+	+	+	–	+	+
	OPS 3	+	+	+	+	+	–	+	+	–	–

“+” indicate positive response, “–” indicate negative response. “U” indicate utilization, “F” indicate fermentation. *Fermentation of sugars was screened in Davis and Mingiolis medium supplemented with 0.1% bromothymol blue and 1% sugar.

### 3.3. Antibiotic sensitivity profile

Antibiotic sensitivity pattern of the endophytic bacterial isolates was determined by disc-diffusion method against six different antibiotics (penicillin G, streptomycin, sulphatriad, tetracycline, ampicillin and chloramphenicol). The bacterial endophytes from leaf lamina, petiole, rhizome and spike tissues of *O. reticulatum* L. were all sensitive to chloramphenicol. One rhizome endophyte, OPR 2 was sensitive to all 6 antibiotics tested. Most of the isolates were also sensitive to tetracycline and resistant to ampicillin and penicillin G (Table 5).

### 3.4. Evaluation of plant growth promoting traits

When grown in tryptophan broth, the endophytic bacterial isolates showed the production of IAA as revealed by the development of pink colour on treatment with Salkowski's reagent (Table 6). Among the 20 isolates, 11 showed IAA production. The concentration of IAA ranged between 5 µg/ml to 39.79 µg/ml. Isolate OPR 7 was the best producer (39.79 µg/ml) followed by OPR 6 (10 µg/ml).

**Table 5.** Screening of bacterial endophytes isolated from leaf lamina, petiole, rhizome and spike tissues of *O. reticulatum* L. for their antibiotic susceptibility following disc-diffusion assay.

Plant part		Bacterial isolate		Antibiotics																						
				Penicillin G (1 unit)				Streptomycin (10 µg)				Sulphatriad (300 µg)				Tetracycline (25 µg)				Ampicillin (10 µg)				Chloramphenicol (25 µg)		
				**Dia^a^ (mm)**		**Rs^b^**		**Dia^a^ (mm)**		**Rs^b^**		**Dia^a^ (mm)**		**Rs^b^**		**Dia^a^ (mm)**		**Rs^b^**		**Dia^a^ (mm)**		**Rs^b^**		**Dia^a^ (mm)**		**Rs^b^**
Leaf lamina		OPL 1		10.3 ± 0.57		R		17.0 ± 1.00		S		18.0 ± 1.00		S		16.5 ± 0.50		I		9.3 ± 0.57		R		24.8 ± 0.76		S
		OPL 2		13.6 ± 0.57		R		22.0 ± 1.00		S		25.6 ± 0.57		S		20.0 ± 1.00		S		13.6 ± 0.57		R		26.0 ± 1.00		S
		OPL 3		25.0 ± 1.00		R		21.3 ± 0.57		S		27.3 ± 0.57		S		24.3 ± 0.57		S		20.0 ± 1.00		R		26.3 ± 0.57		S
		OPL 4		10.6 ± 0.57		R		20.6 ± 0.57		S		25.0 ± 1.00		S		21.6 ± 0.57		S		–		R		26. ± 0.57		S
		OPL 5		27.3 ± 0.57		I		27.5 ± 0.50		S		22.5 ± 0.50		S		25.0 ± 1.00		S		19.6 ± 0.57		R		23.0 ± 1.00		S
Petiole		OPP 1		9.3 ± 0.57		R		20.0 ± 1.00		S		32.3 ± 0.57		S		20.5 ± 0.50		S		12.3 ± 0.57		R		22.8 ± 0.76		S
		OPP 2		–		R		13.3 ± 0.57		I		–		R		19.6 ± 0.57		S		–		R		27.6 ± 0.57		S
		OPP 3		9.6 ± 0.57		R		13.3 ± 0.57		I		–		R		27.3 ± 0.57		S		8.3 ± 0.57		R		28.0 ± 1.00		S
		OPP 4		30.0 ± 1.00		S		21.6 ± 0.57		S		–		R		22.8 ± 0.76		S		7.3 ± 0.57		R		29.3 ± 0.57		S
Rhizome		OPR 1		–		R		23.0 ± 1.00		S		23.5 ± 0.50		S		21.3 ± 0.57		S		–		R		27.8 ± 0.76		S
		OPR 2		40.8 ± 0.76		S		32.3 ± 0.57		S		36.3 ± 0.57		S		26.0 ± 1.00		S		37.6 ± 0.57		S		28.3 ± 0.57		S
		OPR 3		21.0 ± 1.00		R		22.6 ± 0.57		S		–		R		–		R		8.3 ± 0.57		R		20.0 ± 1.00		S
		OPR 4		–		R		20.0 ± 1.00		S		–		R		13.3 ± 0.57		R		–		R		21.0 ± 1.00		S
		OPR 5		25.0 ± 1.00		R		22.8 ± 0.76		S		10.3 ± 0.57		R		21.0 ± 1.00		S		20.6 ± 0.57		R		27.6 ± 0.57		S
		OPR 6		–		R		20.0 ± 1.00		S		19.8 ± 0.76		S		24.3 ± 0.57		S		30.0 ± 1.00		S		25.3 ± 0.57		S
		OPR 7		19.3 ± 0.57		R		9.5 ± 0.50		R		22.0 ± 1.00		S		23.8 ± 0.76		S		18.3 ± 0.57		R		31 3 ± 0.50		S
		OPR 8		–		R		13.3 ± 0.57		I		–		R		20.0 ± 1.00		S		7.6 ± 0.57		R		25.5 ± 0.50		S
Spike		OPS 1		–		R		13.0 ± 1.00		I		22.3 ± 0.57		S		20.5 ± 0.50		S		8.6 ± 0.57		R		25.0 ± 1.00		S
		OPS 2		31.8 ± 0.76		S		24.0 ± 0.50		S		29.6 ± 0.57		S		23.3 ± 0.57		S		25.0 ± 1.00		R		35.0 ± 1.00		S
		OPS 3		20.3 ± 0.57		R		14.6 ± 0.57		I		24.0 ± 1.00		S		21.6 ± 0.57		S		14.3 ± 0.57		R		25.3 ± 0.57		S

^a^Diameter of inhibition zone (mm), ^b^Response to the antibiotic (R = Resistant, I = Intermediate, S = Sensitive). Values represent mean of triplicate readings ± SD.

**Table 6.** Growth associated production of IAA by the endophytic bacteria isolated from leaf lamina, petiole, rhizome and spike tissues of *O. reticulatum* L.

Plant tissue	Bacterial isolate	Growth OD at 540nm	Production of IAA (µg/ml)
Leaf lamina	OPL 2	1.43 ± 0.03	6.12 ± 0.02
	OPL 5	1.46 ± 0.01	5.00 ± 0.10
Petiole	OPP 1	0.49 ± 0.01	7.14 ± 0.03
	OPP 2	1.04 ± 0.01	7.14 ± 0.05
	OPP 4	1.45 ± 0.01	6.12 ± 0.02
Rhizome	OPR 4	1.29 ± 0.01	7.14 ± 0.02
	OPR 5	1.97 ± 0.00	6.12 ± 0.05
	OPR 6	1.53 ± 0.01	10.00 ± 0.05
	OPR 7	1.59 ± 0.02	39.79 ± 0.01
	OPR 8	1.42 ± 0.01	5.00 ± 0.02
Spike	OPS 1	1.34 ± 0.01	8.16 ± 0.02

*Production of IAA was assessed by Salkowski colorimetric assay at 540 nm. Amount of IAA produced was determined from the standard curve of IAA. Values represent mean of triplicate readings ± SD.

**Figure 2.**
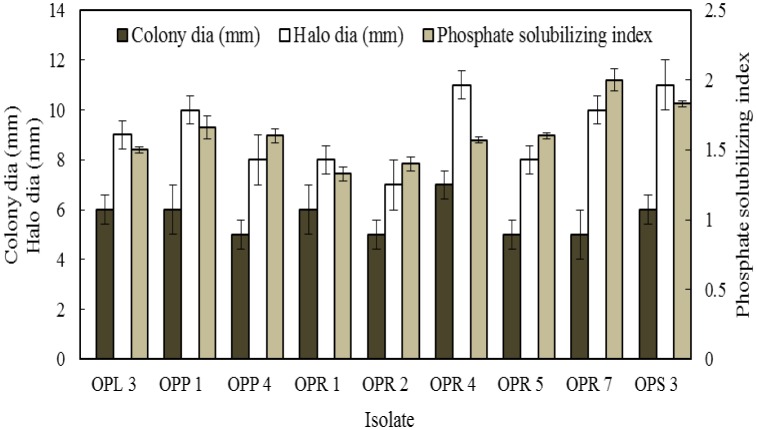
Phosphate solubilizing ability of the bacterial endophytes isolated from *O. reticulatum* L.

The ability of the endophytic bacterial isolates to solubilize insoluble phosphate was detected in 9 isolates as revealed by the formation of clear zone surrounding the growth of the isolates on Pikovskaya's medium. Isolates OPR 7 and OPS 3 showed comparatively higher phosphate solubilizing index (Figure 2).

Out of the 20 isolates inoculated in Norris N_2_-free glucose medium, majority of them could grow in the absence of nitrogen in the medium except OPL 1, OPL 3, OPL 5, OPR 3 and OPS 1. Qualitative siderophore assay showed that the isolates OPL 3 and OPR 4 were good producers of siderophore whereas OPL 4, OPP 1, OPR 1, OPR 7 and OPR 8 were moderate producers (Table 7).

**Table 7.** Growth in N_2_-free medium and production of siderophore by endophytic bacterial isolates of *O. reticulatum* L.

Plant tissue	Bacterial isolate	Growth in N_2_-free medium	Production of siderophore
Leaf lamina	OPL 1	–	–
	OPL 2	+	–
	OPL 3	–	+++
	OPL 4	+	+
	OPL 5	–	–
Petiole	OPP 1	+	++
	OPP 2	+	–
	OPP 3	+	–
	OPP 4	+	–
Rhizome	OPR 1	+	++
	OPR 2	+	–
	OPR 3	–	–
	OPR 4	+	+++
	OPR 5	+	–
	OPR 6	+	–
	OPR 7	+	+
	OPR 8	+	+
Spike	OPS 1	–	–
	OPS 2	+	–
	OPS 3	+	+++

“+”indicate positive response, “–” indicate negative response.

### 3.5. Time course of growth and production of IAA

**Figure 3.**
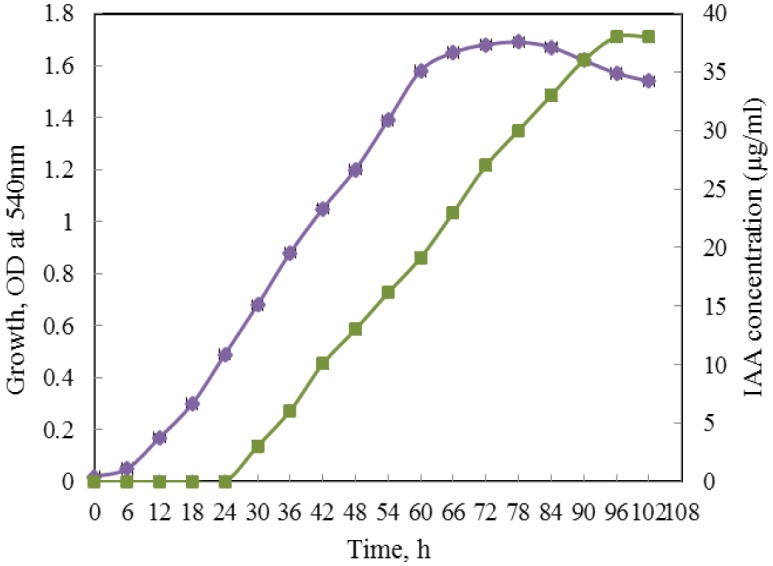
Time course of growth (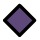) and IAA production (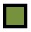) by the bacterial endophyte *Bacillus* OPR 7 isolated from the rhizome of *O. reticulatum* L. *Isolate OPR 7 was grown in tryptophan broth under continuous shaking. Samples withdrawn at every 6 h interval were evaluated for growth (OD at 540 nm). IAA production was assayed by the conventional Salkowski colorimetric assay for each time interval and the concentration of IAA produced was determined from the standard curve. Values represent mean of triplicate readings ± SD.

Isolate *Bacillus* OPR 7, the best IAA producing isolate was chosen for time course study of growth and IAA production in tryptophan broth under batch culture. Production of IAA was initiated in the exponential phase of growth and reached its maximum after 96 h of growth during which it produced nearly 40 µg/ml of IAA (Figure 3).

## 4. Discussion

It has been seen in recent years that plant growth promoting endophytes have the ability to colonize the interior tissues of the host plant and further build a beneficial symbiotic association with their host plants to improve host plant growth [21],[22]. In this study we isolated a total of 20 morphologically distinguishable endophytic bacteria from different parts of *O. reticulatum* L. (Figure 1).The colonization frequency was recorded to be highest in the rhizome and the Shannon-Weaver diversity index also indicated the presence of most diverse types of endophytic bacteria in the rhizome of *O. reticulatum* L. (Table 1). The endophytes so far isolated and characterized were tentatively assigned to the genera *Bacillus*, *Pseudomonas* and *Staphylococcus* (Table 2). Although during the present study, the identity of the bacterial endophytes could not be determined at the species level, which requires an indepth 16S rRNA sequence analysis. *Bacillus* sp. has been reported as an endophyte of *Polygonum cuspidatum* [23], tomato [24] and *Aquilaria* sp. [25]. Similarly, *Bacillus* sp. and *Pseudomonas* sp. have been extensively reported to colonize the roots of many crop plants [26] and found to induce the growth in green gram plants [27]. Species of *Staphylococcus* as endophytes are also not uncommon [28].

Hydrolytic enzymes of endophytes in general appear to be important for the colonization of plant roots [29],[30]. Bacterial endophytes of *O. reticulatum* L. in this study were found to produce a variety of hydrolytic enzymes such as catalase, caseinase, gelatinase, PHB depolymerase, amylase and inulinase (Table 3). The presence of nitrate reductase in some of the isolates suggests that they play a role in the nitrogen cycle, thereby having agricultural and environmental implications. Majority of the isolates could also ferment glucose, sucrose and fructose and were resistant to cell wall inhibiting antibiotics ampicillin and penicillin G (Table 4 and 5). The isolates also possessed multiple plant growth promoting traits such as IAA production, phosphate solubilization, growth in N_2_-free medium and production of siderophore. More than 50% of the endophytic isolates showed IAA production (in the presence of tryptophan) with isolate *Bacillus* OPR 7 being the best producer (Table 6). IAA is reported to increase root size and spreading, resulting in greater nutrient absorption from the soil [31]. Reports of IAA production by plant associated bacteria are not uncommon. *Pseudomonas stutzeri* isolated from *Echinacea* sp. produces 18.8 µg/ml of IAA [32], *Methylobacterium* sp. from red and white clover produces 6–13.3 µg/ml IAA [33] and *Bacillus thuringiensis* produces 1.53–9.71 µg/ml IAA [34].

It is known that improved phosphorous nutrition enhances the overall growth of the plants and help in root development [35]. Nearly 50% of isolates exhibited the phosphate solubilizing activity by forming clear zones (Figure 2). *Bacillus* sp., *Pseudomonas* sp., *Serratia* sp. and *Enterobacter* sp. are reported to solubilize the insoluble phosphate compounds and assist in plant growth [36],[37]. Siderophore production was recorded in 40% of the isolates (Table 7), which are likely to play an important role in the acquisition of nutrients such as iron availability to the plant [38]. Majority of the isolates (15 out of 20 isolates) were able to grow in N_2_-free medium (Table 7) indicating their ability to fix atmospheric nitrogen. For time course study of growth and IAA production the isolate *Bacillus* OPR 7 was chosen and the maximum production was observed after 4 days of incubation (Figure 3). Experiments related to exploration of more bacterial traits for growth promotion are required to further strengthen the findings and application in plant growth and development in a sustainable manner.

## 5. Conclusion

Endophytic bacterial isolates was found to be associated with leaf lamina, petiole, spike and rhizome of *Ophioglossum reticulatum* L. The endophytes produced several hydrolytic enzymes of commercial importance and also possessed plant growth promoting traits. To our knowledge this is the first report addressing the exploration of the diversity of the endophytic bacteria from *O. reticulatum* L. and their evaluation of plant growth promoting traits. These potent endophytic bacteria, either singly or in combination could be developed as an eco-friendly biofertilizer for growth and development of many important plant species including *O. reticulatum* L.
